# A complex network approach to analyse pre-trained language models for ancient Chinese

**DOI:** 10.1098/rsos.240061

**Published:** 2024-05-15

**Authors:** Jianyu Zheng, Xin'ge Xiao

**Affiliations:** ^1^ Department of Chinese Language and Literature, Tsinghua University, Beijing 100084, People's Republic of China; ^2^ Research Center for Language and Language Education, Central China Normal University, Wuhan 430079, People's Republic of China

**Keywords:** ancient Chinese, complex networks, SikuBERT, attention head, language model

## Abstract

Ancient Chinese is a splendid treasure within Chinese culture. To facilitate its compilation, pre-trained language models for ancient Chinese are developed. After that, researchers are actively exploring the factors contributing to their success. However, previous work did not study how language models organized the elements of ancient Chinese from a holistic perspective. Hence, we adopt complex networks to explore how language models organize the elements in ancient Chinese system. Specifically, we first analyse the characters’ and words’ co-occurrence networks in ancient Chinese. Then, we study characters’ and words’ attention networks, generated by attention heads within SikuBERT from two aspects: static and dynamic network analysis. In the static network analysis, we find that (i) most of attention networks exhibit small-world properties and scale-free behaviour, (ii) over 80% of attention networks exhibit high similarity with the corresponding co-occurrence networks, (iii) there exists a noticeable gap between characters’ and words’ attention networks across layers, while their fluctuations remain relatively consistent, and (iv) the attention networks generated by SikuBERT tend to be sparser compared with those from Chinese BERT. In dynamic network analysis, we find that the sentence segmentation task does not significantly affect network metrics, while the part-of-speech tagging task makes attention networks sparser.

## 1. Introduction

The ancient Chinese (classical Chinese), known as ‘wén yán’, remained as the primary written language until the twentieth century [1]. Ancient Chinese people used this writing system to chronicle their observations and understanding of the world [2]. In the present digital age, the ancient Chinese texts also need to be processed in a modern way, and the development of natural language processing (NLP) technology [3,4] indeed provides the support to this area. Particularly, with the emergence of pre-trained language models [5], many ancient Chinese information processing tasks have been completed very well.

The excellent ability of pre-trained language models in processing ancient Chinese demonstrates its effectiveness in representing the text. Thus, it is necessary to investigate the mechanisms by which these models ‘comprehend’ ancient Chinese. In fact, previous work has explored how pre-trained language models ‘learn’ linguistic knowledge, including probing lexical [6,7], syntactic [8,9] and semantic [10–12] knowledge encoded in the models. However, most of these works focus on probing the knowledge at certain aspects, without adopting a holistic perspective to study how the models simulate human language, or to discern potential patterns in how they organize the elements of ancient Chinese.

In order to study how pre-trained models simulate ancient Chinese from a holistic perspective, it is essential to first admit a fact that language, which has been evolving within the human lineage, is a complex system [13]. In this system, the value of each interdependent term results solely from the simultaneous presence of the others [14]. Ancient Chinese, as such a system, is not an exception. For studying a complex system, such as ancient Chinese, complex networks exactly provide us with an effective method [15]. In terms of complex network theory, the elements are nodes connected to each other through edges [16]. The linguistic elements in ancient Chinese and their relationships can also be represented by complex network analysis [17]. Similarly, the structures of language texts organized by pre-trained language models can also be described and analysed by complex networks, which offers a holistic perspective to study how language models ‘learn’ ancient Chinese.

Therefore, in this work, we use complex networks as the research method to investigate the overall characteristics of characters' and words' networks in ancient Chinese, which are constructed by pre-trained language models. Specifically, we use Chinese pre-trained language models and a human-annotated dataset of ancient Chinese, Ancient Chinese Corpus,^1^ as experimental materials. We first explore the characteristics of the characters’ and words’ co-occurrence networks. Then, we measure how each attention head in a pre-trained language model for ancient Chinese, SikuBERT, organizes the attention networks of ancient Chinese and calculates the similarities with the corresponding co-occurrence networks above. Next, we analyse the similarities and differences between two language models, Chinese BERT and SikuBERT, in organizing attention networks. Finally, we employ the model to complete downstream tasks related to ancient Chinese and summarize the network evolution in terms of various network metrics.

According to the experimental results, we observe small-world properties and scale-free behaviour in co-occurrence networks and most attention networks generated by attention heads in ancient Chinese. Moreover, we note a high similarity, exceeding 80%, between attention networks and their corresponding co-occurrence networks. By comparing networks generated by SikuBERT and Chinese BERT, we notice that the networks generated by SikuBERT tend to exhibit greater density and a broader distribution across each metric. Finally, in our dynamic analysis of attention networks, we find that sentence segmentation has minimal impact on network metrics, whereas part-of-speech tagging leads to a sparser attention network.

Our contributions can be summarized from two aspects. In the theoretical aspect, our work reveals the inherent mechanism of how pre-trained language models organize ancient Chinese from the perspective of complex networks. It has taken us a step closer towards a more comprehensive understanding of deep learning systems and their capabilities in language processing. In practice, we can take complex networks as indicators to evaluate the performance of a language model and excavate some information to design an improved model for ancient Chinese in the future.

## 2. Related works

### 2.1. Natural language processing for ancient Chinese

NLP for ancient Chinese is an emerging field that aims to process ancient Chinese texts automatically. Some work focused on constructing the relevant datasets, such as Zhou *et al*.’s evaluation benchmark for classical Chinese [18] that was used to test pre-trained language models by sentence classification, sequence labelling, reading comprehension and machine translation. Pan *et al*. [19] also constructed an ancient Chinese dataset for word sense disambiguation. However, the majority of work is still on designing models and algorithms to process specific tasks in ancient Chinese. For example, Tang & Su [20] proposed a cross-era learning framework called CROSSWISE to segment ancient Chinese sequences of different eras. Zhang *et al*. [21] employed augmentation strategies, including continual pre-training, adversarial training and ensemble learning, to improve the performance of language models on word segmentation and part-of-speech tagging in ancient Chinese. Feng *et al*. [22] designed a model architecture, Bert-ancient-chinese+LSTM+CRF (BAC+RLSTM+ CRF) for named entity recognition in ancient Chinese. Wang *et al*. [23] created a model that included a multi-head attention layer to capture long-distance related features, by extracting entities and their relationships simultaneously. Furthermore, some research has paid more attention to developing pre-trained language models for ancient Chinese, such as SikuBERT, which was created by Wang *et al*. [24] by continuing learning Chinese BERT [5], using *Siku Quanshu* full-text corpus as the training set. Tian *et al*. [25] released AnchiBERT, which was trained on large-scale ancient Chinese corpora, and the experimental results showed that it outperformed Chinese BERT on many tasks related to ancient Chinese.

### 2.2. Complex networks in language system

Complex networks are a powerful tool for understanding and analysing the organization of language systems. Many works used complex networks to gain insights in the language systems. For example, Wachs-Lopes and Rodrigues [26] investigated key properties of language complex networks, such as degree distribution, weight distribution and average clustering coefficient. Li *et al*. [27] used complex network to identify the associative learning mechanism, which plays a crucial role in acquiring vocabulary, particularly in second language (L2) learning. Jiang *et al*. [28] constructed nine networks of English compositions by Chinese students and figured out that L2 syntactic networks also exhibited the scale-free and the small-world properties. Sheng and Li [29] analysed the properties of written English and Chinese with weighted complex networks. Their findings suggested that the two languages might possess different linguistic mechanisms and characteristics. Furthermore, complex networks have been applied in various NLP tasks. Akimushkin *et al*. [30] proposed a methodology based on word co-occurrence networks to identify the authorship of 80 texts. Arruda *et al*. [31] used a complex network model to describe the local topological/dynamical properties of function words, and applied this approach to a text classification task. Matas *et al*. [32] constructed 10 distinct complex networks from Wikipedia entries and extracted domain knowledge with centrality measures.

The previous research has provided us with references regarding the research subjects (pre-trained language models for ancient Chinese) and methods (complex networks). However, most studies [6–12] on probing language models have not comprehensively elucidated the patterns and principles about the organization of linguistic elements within the models. Therefore, we use complex networks to comprehensively study how these pre-trained language models organize linguistic elements at different levels. To the best of our knowledge, our work is among the first to utilize complex networks to study how pre-trained language models organize linguistic elements in ancient Chinese.

## 3. Theoretical background

### 3.1. Complex networks

Complex networks refer to a type of network with a large number of interconnected elements [15], where elements can be represented as nodes, and the connections between them are denoted by edges [33]. By modelling a system with complex network, its structural properties, dynamics and emergent behaviours can be studied. In this way, we can better understand the complex relationships and patterns within these interconnected systems [34,35].

Formally, a complex network can be represented as ***G*** = (***V***, ***E***), where ***V*** and ***E*** denote the set of nodes and the set of edges, respectively. To analyse the properties of complex networks, various metrics are proposed. In our experiments, we adopt the following complex network metrics:

— ***N***: number of nodes, representing the quantity of tokens in a corpus.
— ***E***: number of edges, indicating the connections generated between tokens in the corpus.
— ***<k>***: average degree, measuring a token’s average capability to combine with other tokens.
— ***L***: average shortest path length, assessing the efficiency of information transfer between nodes.
— ***C***: clustering coefficient, reflecting the density of the network to some extent.
— ***D***: diameter, offering insight into the scale of the network to some extent.
— ***Bc***: between centrality, used to evaluate the importance of a node.
— ***Ec***: eigenvector centrality, embodying the importance of a node.

Furthermore, two common properties frequently observed in complex networks are as follows.

— *The small-world property*: the average shortest path length between nodes is relatively short.
— *The scale-free behaviour*: the distribution of node degrees follows a power-law distribution.

### 3.2. Attention heads in BERT

Bidirectional encoder representations from transformers (BERT) [5] is a language model based on the transformer architecture. It is pre-trained on a massive corpus of text, including English Wikipedia and BooksCorpus [36]. During pre-training BERT, two primary tasks are implemented: predicting randomly masked words in the input (MLM) and classifying whether a sentence follows another sentence in the corpus (NSP). The two tasks enable BERT to understand language nuances, so that it can achieve state-of-the-art performance on a variety of NLP tasks.

Within BERT, the attention heads are facilitated by the attention mechanism, which plays a crucial role in capturing relationships between different tokens in a sentence. The attention heads allow BERT to analye the importance of different tokens in a sentence. Next, we will take ancient Chinese sentences as input and explain their working mechanism.

**Step 1: transforming input**

Given an ancient Chinese sentence *s* = *c*_1_, *c*_2_, ..., *c*_*n*_ , where *c*_*i*_ is a token in the sentence, BERT transforms each token *c*_*i*_ into a corresponding vector representation *e*_*i*_ through an embedding layer, which greatly facilitates subsequent calculations.

**Step 2: obtaining query, key, and value vectors**

In this step, a linear function is employed to map each vector *e*_*i*_ into query (*q*_*i*_), key (*k*_*i*_) and value (*v*_*i*_) vectors, with trainable parameters *W*_*q*_, *W*_*k*_ and *W*_*v*_


(3.1) $$q_{i},k_{i},v_{i}=W_{q}*e_{i},W_{k}*e_{i},W_{v}*e_{i}$$

Through this step, different representations of each vector *e*_*i*_ can be generated, which can be applied to capture different aspects of its context

**Step 3: computing attention scores**

For each token in the input, an attention head computes the attention score *α* about the token relative to other tokens in the input, which is the dot product of the query vector of the current token with the key vectors of all other tokens, followed by a scaling operation to stabilize the gradients. To focus more on the most relevant tokens in the input, a softmax function is then applied to the attention scores for each token


(3.2) $$\alpha_{ij}=soft\text{max}\left(\frac{q_{i}^{T}k_{j}}{\sqrt{d}}\right),$$

where $\sqrt{d}$ is a scaling factor.

The attention score *α* can determine the importance or relevance of each token in the sentence of other tokens.

**Step 4: weighted summing values**

In this step, an output vector *h*_i_ is generated as a weighted sum of the value vectors based on the attention distribution *α*


(3.3) $$h_{i}=\sum_{j=1}^{n}\alpha_{ij}v_{j}.$$

The output vector *h*_*i*_ represents the contextual representation of each token *c*_*i*_, where tokens with higher attention scores have a more significant influence on the final representation. In other words, this step allows BERT to capture the most relevant information from other tokens.

**Step 5: generating multi-head self-attention**

Within BERT, the self-attention process (Steps 1–4) is performed multiple times in parallel, each with its own set of query–key–value vectors and attention scores. These parallel self-attention mechanisms are referred to as ‘attention heads’. The outputs from all the attention heads in the same layer could be concatenated to produce the final contextual representation ${\hat{h}}_{i}$ for token *c*_*i*_.


(3.4) $${\hat{h}}_{i}=[h_{i}^{1},h_{i}^{2},\ldots ,h_{i}^{n}],$$

where $h_{i}^{j}$ represents the hidden state of *j*th head of token *i*. The hidden representation ${\hat{h}}_{i}$ would be then passed into the next layer of BERT for the same operation.

The multi-head self-attention mechanism (‘attention heads’) allows BERT to encode various linguistic information and context, including dependency relations [8,37], semantic roles [38,39], as well as recognizing negation and understanding scope in linguistics [40,41], so that has significantly promoted BERT as a powerful tool for various NLP tasks.

## 4. Experiment and analysis

**Figure 1.**
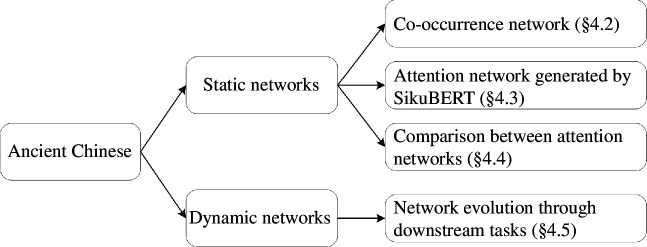
The illustration of experiment organization. It consists of the analysis of static network and dynamic network.

In this section, we first introduce the dataset and models to be used (§4.1). Next, we implement our experiments from two aspects: both static and dynamic network analyses, as displayed in figure 1.

For the analysis of static networks, what we explore including co-occurrence networks of ancient Chinese, attention networks generated by SikuBERT and comparisons between attention networks. Specifically, since no prior research has ever investigated the complex networks of ancient Chinese, we construct characters’ and words’ co-occurrence networks and study their properties (§4.2). In contrast to human language networks, we subsequently study the attention networks of ancient Chinese generated by SikuBERT (§4.3), including exploring the properties of these attention networks (§4.3.1), investigating the networks generated by some special heads which capture positional relationships between words (§4.3.2) and exploring the network properties across layers (§4.3.3). Furthermore, in order to demonstrate the advantage of SikuBERT in processing ancient Chinese compared with other language models from a complex network perspective, we compare the attention networks generated by SikuBERT and Chinese BERT (§4.4).

Regarding the experiments on dynamic networks, we use a typical language model for ancient Chinese, SikuBERT, to perform different tasks and observe the network evolution in terms of network metrics.

### 4.1. Dataset and models

The Ancient Chinese Corpus^1^ (ACC) is adopted as dataset in our experiments. The development of this corpus was carried out by Nanjing Normal University. It contained word-segmented and part-of-speech tagged text from Zuozhuan, a classic Chinese work that was thought to date back to the Warring States Period, from 475 to 221 BC. The Ancient Chinese Corpus comprised a total of 180 000 Chinese characters and 195 000 segments (including words and punctuation). Additionally, the part-of-speech tag set used in the corpus was created by Nanjing Normal University, and it consisted of 17 tags.

In selecting pre-trained language models for our experiment, SikuBERT can serve as a suitable candidate due to its extensive representativeness. Additionally, we believe that the same research methodology can be applied to study other language models in the future. This model is developed by the collaboration of Nanjing Agricultural University and Nanjing Normal University. Specifically, SibuBERT was constructed by continuing training in Chinese BERT [5], with the proofread and high-quality full-text corpus of *Siku Quanshu* as the unsupervised training set. During the continual training of SikuBERT, the masked language modelling (MLM) task was employed, wherein 15% of tokens were randomly masked. This model possesses significant advantages in processing ancient texts. We can access SikuBERT through the following link.^2^

For calculating complex network features in the subsequent sections, we will use the ‘NetworkX’ package in Python software.

### 4.2. Co-occurrence networks of ancient Chinese

**Figure 2.**
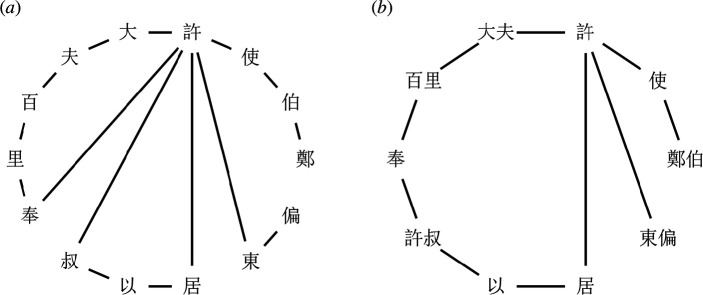
The co-occurrence networks of an example sentence from ACC. (***a***) The characters' co-occurrence network and (***b***) the words' co-occurrence network.

Before studying how language models organize the complex networks of ancient Chinese, we explore the inherent properties of this language network generated by human. Following the previous work [42], we construct characters' and words' co-occurrence networks in ancient Chinese. In the characters' co-occurrence networks, we connect each pair of adjacent characters with an edge in sentences; for the words’ co-occurrence networks, we establish connection between each pair of adjacent words. For illustration, we use the sentence ‘鄭伯使許大夫百里奉許叔以居許東偏’ from ACC to display the co-occurrence networks in figure 2.

**Network property analysis**. After constructing the co-occurrence networks, we measure their properties and present the results in table 1. We discover that the number of nodes (*N*) in the characters' co-occurrence network is significantly lower than that of words’ (3290 versus 11 746), which reflects that the number of common-used characters is much smaller than that of common-used words. On the other hand, the average degree (<*k*>) of characters' network is substantially higher than that of the words’ network (14.16 > 4.37). It ultimately leads to that the difference in the number of edges (*E*) between these two networks is not very significant (46 597 and 51 347). This might be because that ancient Chinese characters are more flexible when combined with other linguistic elements. Hence, the average degree of the characters' network is larger. However, the combination of words is constrained by syntax and pragmatics, so that the average degree of the words’ network is smaller. Furthermore, we observe that the average shortest distances (*L*) of both networks are in the range of 2–3, signifying that any two characters or words can be connected through very few nodes in the networks. This phenomenon also reflects that ancient Chinese people could organize different characters or words together through very few steps, which was very advantageous for information transmission.

**Table 1.** The major properties of characters' or words’ co-occurrence networks in ACC.

	*N*	*E*	*<k* ***>***	*L*	*C*	*D*	*Bc*	*Ec*	*L*_random_	*C*_random_
character	3290	46 597	14.16	2.58	3.76e-1	7	4.79e-4	8.94e-3	2.77	8.74e-3
word	11 746	51 347	4.37	3.06	2.82e-1	8	1.71e-4	3.47e-3	4.57	6.38e-4

**Figure 3.**
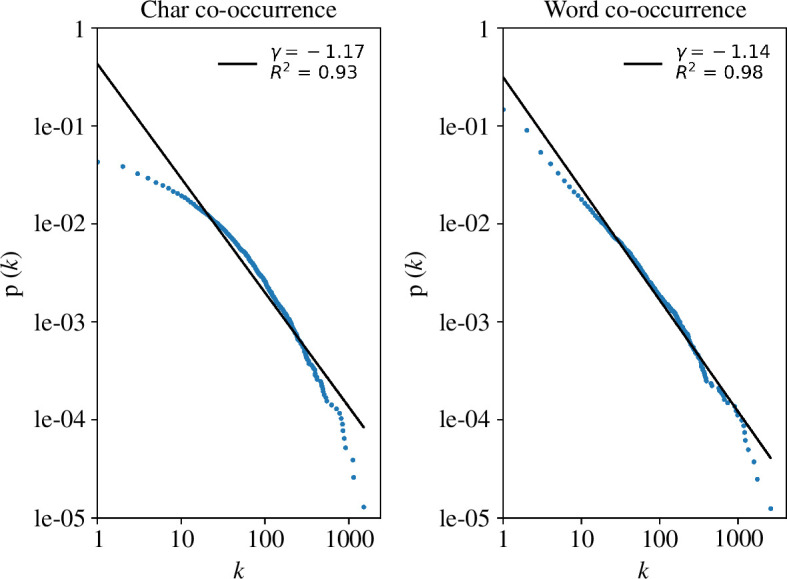
Cumulative degree distributions of co-occurrence networks in ACC. The left part represents the character co-occurrence network, while the right part represents the word co-occurrence network. Both networks adhere to power-law distributions with significant fitting coefficients.

**Small-world properties and scale-free behavior**. To explore whether the two co-occurrence networks exhibit small-world properties and scale-free behavior, we construct two random networks with the same number of nodes and edges to compare. The values in average shortest path length (*L*) and clustering coefficient (*C*) for random networks are displayed in *L*_random_ and *C*_random_ columns of table 1. According to table 1, we could easily find that both co-occurrence networks exhibit the small-world properties, where *L* ≈ *L*_random_ and *C* >> *C*_random_. This implies that ancient Chinese people are able to quickly mention any two characters or words during communication, thereby ensuring fast information dissemination. Small-world properties also reflect the evolution of ancient Chinese system follows ‘the principle of least effort’ [43]. Besides, we also fit the cumulative degree distributions of the two co-occurrence networks and observe that they both exhibit scale-free behaviour, as shown in figure 3, where *γ* represents the power-law exponent and *R* ^2^ is the fitting coefficient. Notably, *γ* falls within the range of 1–2, and *R* ^2^ is also highly significant. The results suggest that the collocation ability of a character or a word is inversely proportional to its proportion in the entire vocabulary. Only a small number of characters and words exhibit strong combination capabilities, which are also commonly used in ancient Chinese.

### 4.3. Attention networks of ancient Chinese generated by SikuBERT

**Figure 4.**
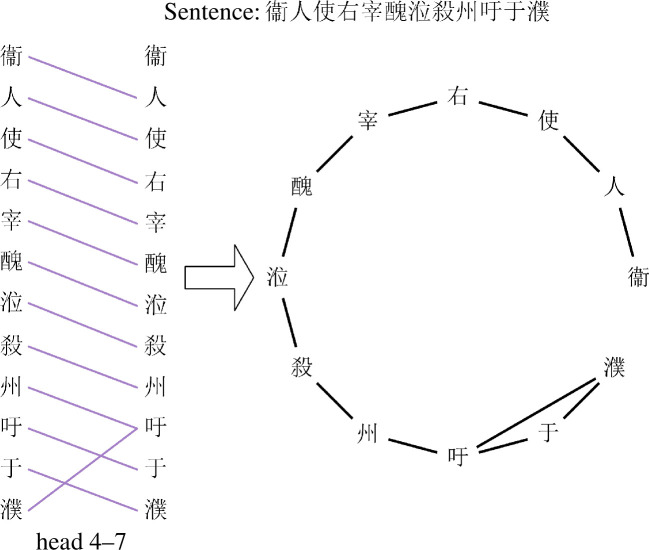
An illustration about constructing a characters’ attention network. Given an example sentence, the left part displays each character and its most attending character in terms of the attention matrix generated by ‘head 4–7’. And the right part presents an attention network based on the attention relationships between characters.

As mentioned in §3.2, the attention heads within SikuBERT can generate the attention relationships among characters. Hence, to investigate the organization of ancient Chinese in SikuBERT, we adopt the information provided by each attention head. Specifically, when a sentence is input into SikuBERT, each head generates an attention matrix between characters of this sentence. Then, we take each character of this sentence as node and connect an edge between each character and its most attending character. By this way, a character’s attention network can be constructed. Figure 4 illustrates the construction process of the attention network with an example sentence from ACC. In figure 4, the left part shows each character and its most attending character according to the attention matrix generated by the seventh head in the fourth layer, namely ‘head 4–7’. The right part displays the resulting attention network for characters. Furthermore, when constructing the words’ attention network, we first adopt the word segment in ACC as the standard, and then sum the columns and average the rows corresponding to the constituent characters of the standard words in the attention matrix, so that a word attention matrix can be obtained. Then, we follow the same procedure as we do for characters to create words’ attention networks.

#### 4.3.1. The properties of attention networks

**Figure 5.**
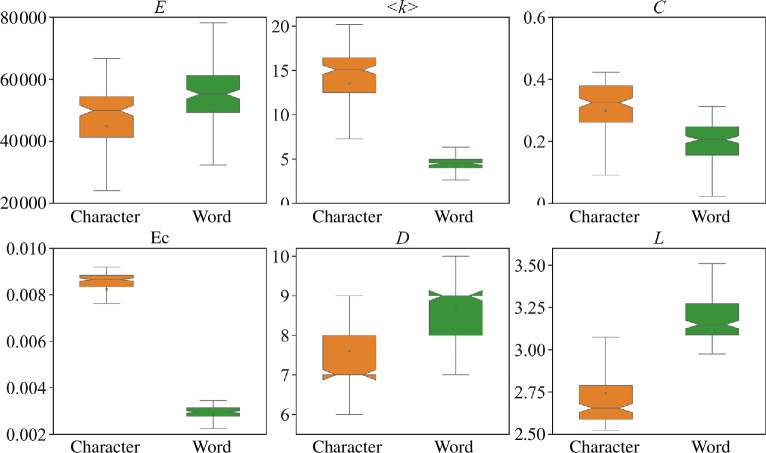
The properties of attention networks in ACC. Boxplots are employed to illustrate the distribution of all networks across various network metrics. Each orange boxplot denotes a distribution of characters’ attention networks, and each green boxplot signifies a distribution of words’ attention networks.

**Network property analysis**. After generating characters’ and words’ attention networks of each attention head, we analyse their network properties. The results are displayed in figure 5, where each subplot shows a specific network property for characters’ and words’ attention networks, respectively. In figure 5, we find that the distributions of characters’ networks in degree (<*k*>), eigenvector centrality (***Ec***) and clustering coefficient (*C*) are higher than those of words’ networks. This observation suggests that the attention heads organize characters of ancient Chinese in a more flexible way, resulting in denser characters’ attention networks. Notably, the distribution of character’s attention network in the metric eigenvector centrality (***Ec***) is significantly higher than that of word’s attention network. This discrepancy can be attributed to the above-mentioned fact that the number of characters is lower than the number of words, resulting in denser networks for characters. Consequently, the importance of nodes within character’s networks is more evident. Furthermore, the metrics such as average shortest path length (*L*) and diameter (*D*) reveal that characters' attention networks exhibit lower values compared with those of words, implying that characters’ attention networks are of smaller scale. Consequently, characters tend to connect with other characters with shorter steps.

**Small-world properties and scale-free behavior**. Furthermore, we assess the small-world property of these attention networks and find that most of the attention networks (141/144) display this property. It indicates that most of the attention heads connect any two words or characters with very few steps while organizing the attention networks, so that the relationships between any characters or words can be better processed by the BERT model. However, there exist three heads deviating from this property in organizing characters’ and words’ attention networks, namely head 3–7, head 4–3 and head 5–3. The property results of the networks generated by the three heads are displayed in table 2. By scrutinizing the attention relationships generated by these heads, we find that they primarily allow each character or word to focus on itself most of time. Consequently, most nodes remain isolated, and very few edges appear in the attention networks. Additionally, in investigating scale-free behaviour, we find that most of networks still exhibit the behaviour, reflecting that most of attention heads tend to enable a small proportional of characters or words to establish more connections with other characters or words. However, there are exceptions, such as head 3–7 and head 4–3. The power-law exponents of the attention networks formed by the two heads are lower than −3, failing to meet the requirements for scale-free behaviour.

**Table 2.** Properties of attention networks in ACC from three under-performed heads.

head	network	*N*	*E*	<*k* **>**	*γ*	*R* ^*2*^	*L*	*C*	*L*_random_	*C*_random_
3-7	character	3310	3378	1.02	−4.16	0.75	1.09	0	10.24	4.68e-4
	word	12 341	12 558	1.02	−3.20	0.87	1.97	0	12.26	1.56e-4
4-3	character	3310	3441	1.04	−4.57	0.88	1.50	0	10.30	1.03e-3
	word	12 341	12 496	1.01	−4.12	0.90	1.58	0	12.36	1.03e-4
5-3	character	3310	3863	1.17	−1.98	0.95	4.30	2.08e-3	9.25	1.18e-3
	word	12 341	12 769	1.03	−2.53	0.93	4.40	1.79e-4	12.19	0

**Figure 6.**
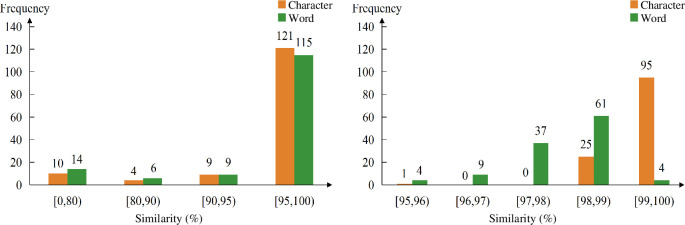
The similarity distribution between the attention networks and the co-occurrence networks. The left section presents the statistics of similarity spanning from 0 to 100; and the right section denotes the detailed statistics of similarity within the range from 95 to 100. Moreover, the orange bar and the green bar correspond to the number of characters’ attention networks and words’ attention networks within each similarity range, respectively.

Based on the analysis above, we can affirm that most of attention networks in SikuBERT display the small-world properties and scale-free behaviour in organizing ancient Chinese text. Hence, we continue to explore the extent of the similarities between the attention network and the corresponding co-occurrence network. We normalize all the seven metrics (except for *N*) to calculate the similarities, and the results are displayed in figure 6. The left part in figure 6 reflects that over 80% of attention networks lie in [95,100) in terms of the similarity distribution. This suggests that the way attention heads organize the language system is very similar to that of humans. In order to observe more detail, we display the specific distribution of the interval [95, 100) at the right part of figure 6, where the similarities of words’ attention networks mostly lie in [97,99), while the results for characters’ reside in [99, 100) absolutely. These results suggest that attention heads are better at ‘learning’ the organizational structure between characters.

**Figure 7.**
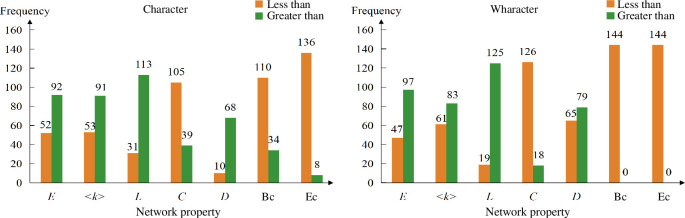
Comparison between attention networks with the corresponding co-occurrence networks. The left part displays the comparison results between characters’ attention networks and the character co-occurrence network; while the right part presents the results between words’ attention networks and the word co-occurrence network. Each orange bar and green bar delegate the number of attention networks that are less than and greater than the corresponding co-occurrence network, respectively.

Furthermore, we compare each attention network with the corresponding co-occurrence network in terms of various network metrics, as shown in figure 7. We observe that the values of most attention networks exceed those of the corresponding co-occurrence networks in some metrics, such as the number of edges (*E*) and the average degree (<*k*>), which reflects that the majority of attention networks can exhibit greater diversity compared with human language network, when organizing various language components. On the other hand, the values of co-occurrence networks are larger than those of most attention networks in the metrics such as clustering coefficient (*C*), betweenness centrality (***Bc***) and eigenvector centrality (***Ec***), suggesting that the co-occurrence networks are denser. This also implies that the connections between language components in most attention networks are not as closely linked as those in human language network.

#### 4.3.2. Network properties and positional relationships

From §4.3.1, we have found that each attention network generated by the corresponding head possesses distinctive characteristics. Considering that many attention heads are designed to comprehend language, they pay close attention to the positional relationships between characters or words. Therefore, we explore the properties of attention networks generated by these heads.

**Figure 8.**
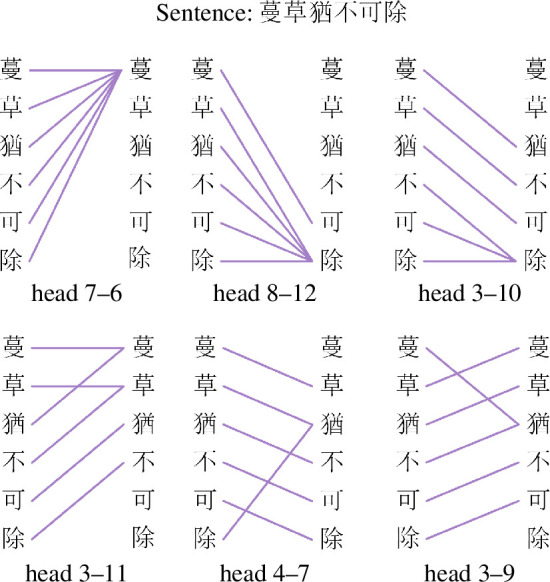
An example sentence demonstrating attention relationships between characters. These relationships are generated by various heads adept at capturing different positional relationships effectively.

**Figure 9.**
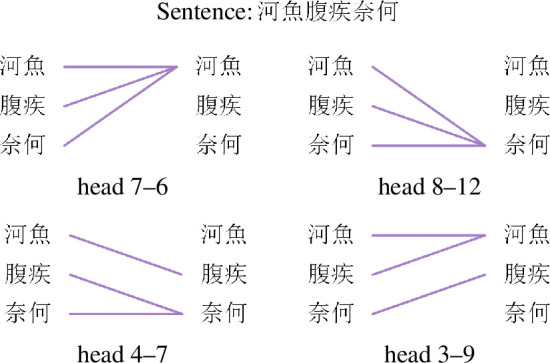
An example sentence demonstrating attention relationships between words. These relationships are generated by various heads adept at capturing different positional relationships effectively.

We first identify some specific attention heads that can capture positional relationships well, as shown in table 3. The ‘acc’ column is the accuracy of each head in capturing the corresponding positional relationship. According to table 3, there indeed exist some heads that could capture some positional relationships well, even with the learning performance reaching over 98%. For better understanding, we use figures 8 and 9 to illustrate the attention mechanisms between characters or words from these heads by an example sentence.

**Table 3.** Some special heads capturing positional relationships.

type of relationships	positional relationships	head	acc (%)
relationships between characters	attending to the first character	7–6	70.83
	attending to the last character	8–12	60.74
	attending after a character	3–10	99.15
	attending before a character	3–11	98.79
	attending to the next character	4–7	99.77
	attending to the previous character	3–9	99.99
relationships between words	attending to the first word	7–6	78.18
	attending to the last word	8–12	66.82
	attending to the next word	4–7	88.62
	attending to the previous word	3–9	90.62

Next, we calculate the properties of the characters’ and words’ attention network corresponding to these heads, as shown in table 4. By analysing, we find that for the head, such as head 7–6 and head 8–12, allowing each character (word) in a sentence to attend to the first character (word) or the last character (word), the attention network generated by this kind of head tends to produce more edges, so that the values of the number of edges (*E*) and the average degree (<*k*>) are larger. Moreover, since only the first or the last character (word) can be connected to the other character (word), any other links between characters or words within the sentence must pass through the first or the last character (word), leading to the shortest path lengths (*L*) of this network being greater. In addition, we also observe that the performance of head 4–7 and head 3–9 in capturing the neighbouring relationships between characters is over 99% in table 3, which indicates that the characters’ networks generated by the two heads are very similar to the characters’ co-occurrence network. After computing the similarity results between the two in terms of network metrics, we find that their similarities are very high, achieving 99.27% (head 3–9) and 99.19% (head 4–7), respectively.

**Table 4.** The properties of attention networks from the heads reflecting positional relationships.

network	head	*N*	*E*	*<k* ***>***	*L*	*C*	*D*	*Bc*	***Ec***
character	7–6	3310	58 228	17.59	2.62	0.38	7	4.76e-4	8.59e-3
	8–12	3310	55 865	16.88	2.62	0.36	8	4.65e-4	8.69e-3
	4–7	3310	50 781	15.34	2.54	0.41	7	4.54e-4	8.95e-3
	3–9	3310	51 532	15.57	2.55	0.41	7	4.57e-4	8.91e-3
	3–10	3310	55 624	16.80	2.59	0.38	8	4.60e-4	8.71e-3
	3–11	3310	55 458	16.75	2.60	0.39	7	4.64e-4	8.65e-3
word	7–6	12 341	65 193	5.28	3.19	0.24	9	1.55e-4	3.00e-3
	8–12	12 341	60 318	4.89	3.16	0.25	8	1.40e-4	3.04e-3
	4–7	12 341	56 149	4.55	3.07	0.22	8	1.16e-4	3.01e-3
	3–9	12 341	56 379	4.57	3.03	0.24	8	1.12e-4	3.07e-3

#### 4.3.3. Network properties across layers

**Figure 10.**
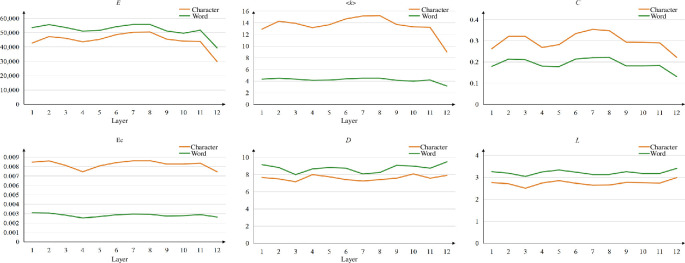
The properties of attention networks across layers, where each value is the average of attention networks generated by the heads from the same layer. The orange and green lines depict the characters’ and words’ attention networks, respectively.

In SikuBERT, the heads in each layer are independent of each other to process the input information, and then the hidden states generated by the heads from the same layer are combined and used as input for the next layer. Hence, it is meaningful to study the attention networks across layers. Figure 10 shows the average of each network metric of the attention networks within the same layer. We can find that these two types of networks have a noticeable gap while their fluctuations remain relatively consistent in each metric, except for the average degree (<*k*>), which reflects that these attention heads follow similar cross-layer patterns when generating both character and word networks. As we move across the layers, the networks tend to become sparser on layer 3–layer 5 and layer 8–layer 12, due to smaller clustering coefficients (*C*). This might be because the networks from these layers hold less diversity in combining characters and words, adopting more fixed patterns to connect nodes.

### 4.4. Comparison between attention networks generated by different models

Compared with other pre-trained language models, such as Chinese BERT [5], SikuBERT has a greater advantage in processing ancient Chinese texts, particularly, in tasks such as word segment and part-of-speech tagging in ancient Chinese. Does this advantage also reflect in the properties of language networks SikuBERT generates? To address this question, we compare the attention networks generated by SikuBERT and Chinese BERT. According to Wang’s work,^3^ SikuBERT is developed by continuing pre-training on Chinese BERT, with the verified high-quality *Siku Quanshu* full-text corpus as the training set. Hence, the corresponding heads in the two BERT models can be compared with each other.

**Figure 11.**
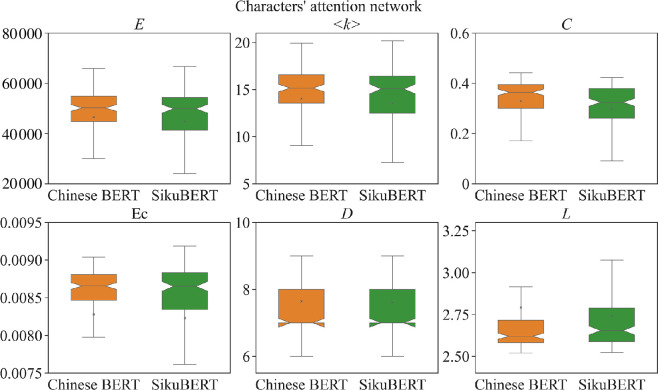
The properties of characters’ attention networks generated by SikuBERT and Chinese BERT. Boxplots are employed to illustrate the distribution of all networks across various network metrics. Orange and green boxplots denote for Chinese BERT and SikuBERT, respectively.

**Figure 12.**
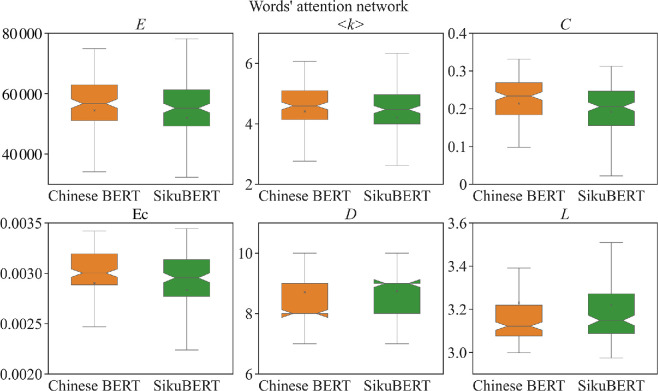
The properties of words’ attention networks generated by SikuBERT and Chinese BERT. Boxplots are used to illustrate the distribution of all networks across various network metrics. Orange and green boxplots denote for Chinese BERT and SikuBERT, respectively.

**Figure 13.**
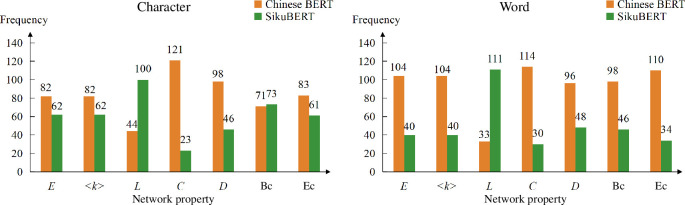
Comparison between attention networks generated by SikuBERT and Chinese BERT. The left part represents the comparison results in characters’ networks, while the right part denotes the results in words’ networks. Orange and green bars display the numbers of networks, which are larger in the corresponding metric generated by Chinese BERT and SikuBERT, respectively.

Figures 11 and 12 show the properties of characters’ and words’ attention networks, respectively. We find that the attention networks generated by SikuBERT tend to be sparser in general. The values of some metrics, such as clustering coefficient (*C*), number of edges (*E*), average degree (<*k*>) and eigenvector centrality (***Ec***) are smaller, while the shortest path length (*L*) is larger. It suggests that the heads in SikuBERT tend to adopt more consistent way to organize attention networks, leading to generating sparser networks. Moreover, we also determine from which BERT each attention network has a larger value in each metric, as shown in figure 13. The results from figure 13 reflect that Chinese BERT has a tendency to generate networks with larger values in most of network metrics. This finding is basically consistent with the results reflected in figures 11 and 12. In addition, figures 11 and 12 also reveal that the results generated by SikuBERT exhibit greater divergence in their distributions. Specifically, SikuBERT has a broader range in each metric when compared with Chinese BERT. This reflects that SikuBERT could possess a more comprehensive understanding of the relationships between characters and words. In conclusion, these findings reflect that the attention heads in SikuBERT could potentially learn the relationships between ancient Chinese components more effectively, thereby surpassing Chinese BERT in various ancient Chinese processing tasks.

### 4.5. Network evolution through downstream tasks

Except analysing the static properties of attention networks, we explore the dynamic changes of networks when SikuBERT completes different downstream tasks. Specifically, we focus on two sequence labelling tasks: sentence segment and Part-of-speech tagging. The sentence segmentation task poses a particular challenge due to the absence of punctuation marks in ancient Chinese, making it difficult to distinguish between individual sentences. In addition, we use the ACC as our experimental corpus, which is divided into training and test sets in a ratio of 9:1. We run SikuBERT for 10 epochs on each downstream task, conducting three times with different random seeds and taking the average results.

**Figure 14.**
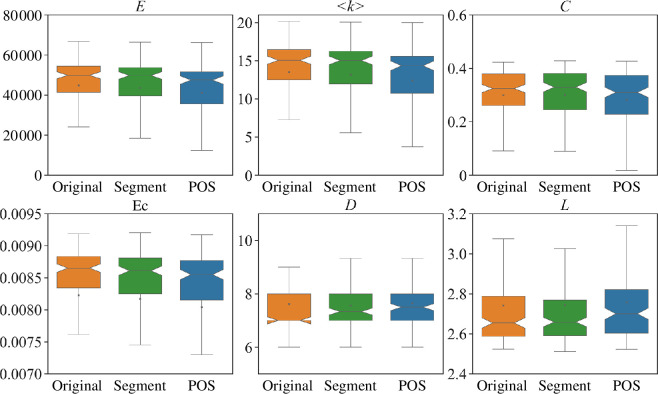
The properties of characters’ attention networks in downstream tasks. The distribution of all networks in each network metric is illustrated by boxplot. The orange boxplot denotes the original distribution of networks, while the green and blue boxplots reflect the distributions of networks after SikuBERT completes sentence segment and part-of-speech tasks, respectively.

**Figure 15.**
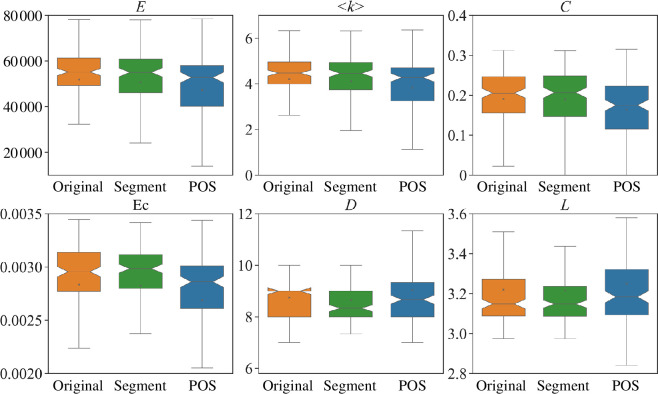
The properties of words’ attention networks in downstream tasks. The distribution of all networks in each network metric is illustrated by boxplot. The orange boxplot denotes the original distribution of networks, while the green and blue boxplots reflect the distributions of networks after SikuBERT completes sentence segment and part-of-speech tasks, respectively.

The experimental results of network changes are displayed in figures 14 and 15. We find that the impact of sentence segmentation on network metrics is not significant. This might be because SikuBERT only needs to identify potential breakpoints within the text sequence. In other words, only learning some local information can finish this task. Consequently, attention networks representing the relationships between characters and words exhibit little change during training. On the other hand, the part-of-speech tagging task has a greater impact on attention networks, with some metrics, such as number of edges (*E*), average degree (*<k>*), eigenvector centrality (***Ec***) and clustering coefficient (*C*) decreasing, while the shortest path length (*L*) increasing. It indicates that the characters’ and words’ attention networks become sparser. The reason for this phenomenon might be that the part-of-speech tagging task requires to learn the neighbouring relationships between words, so the attention networks need to be adjusted, focusing on more frequently occurring relationships to accomplish this task.

**Figure 16.**
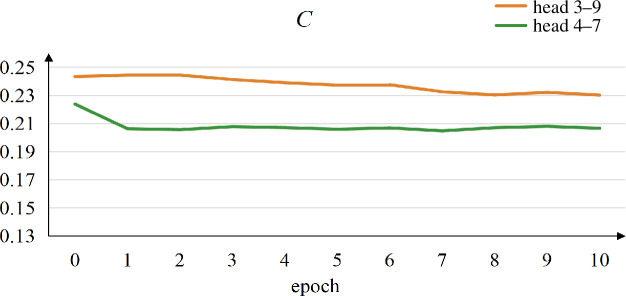
The changes in the clustering coefficient for the words’ attention networks generated by ‘head 3–9’ and ‘head 4–7’, denoted by orange and green lines, respectively.

We also specifically demonstrate the metric changes of some attention networks while completing downstream tasks. As mentioned above, the Part-of-speech tagging task requires SikuBERT to learn the neighbouring relationships between words during training, so we select the two attention heads, ‘head 3–9’ and ‘head 4–7’, that capture the relationships between neighbouring words well, with an accuracy of around 90%. Considering that clustering coefficient serves as a valuable metric for reflecting variations in network density [44], we display the changes in this metric for the words’ attention networks generated by the selected heads, as shown in figure 16. From this figure, it could be seen that the clustering coefficient decreases gradually with the increase of epoch. This also reflects that part-of-speech tagging task makes the attention networks by such heads pay more attention to the neighbouring relationships between words.

## 5. Conclusions

In this article, we study the complex networks of ancient Chinese. Our findings are as follows.

— The characters’ and words’ co-occurrence networks in ancient Chinese demonstrate small-world properties and scale-free behaviour.
— Most of characters’ and words’ attention networks, generated by attention heads within SikuBERT (282/288), also exhibit small-world properties and scale-free behaviour.
— Over 80% of attention networks exhibit high similarity with the corresponding co-occurrence networks, with the level of similarity predominantly falling within the range of [95, 100) in terms of network metrics. Notably, characters’ attention networks exhibit higher similarity compared with the words’ attention networks.
— When investigating attention heads that capture positional relationships well, we observe that the attention networks, whose heads let each character or word in a sentence attend to the first (last) character or word, have a larger number of edges (*E*), the shortest path lengths (*L*) and the average degree (<*k*>).
— In exploring the properties of attention networks across layers, we find that although a noticeable gap exists between characters’ and words’ attention networks, their fluctuations in each metric remain relatively consistent. In addition, the networks tend to become sparser in layer 3–layer 5 and layer 8–layer 12, due to smaller clustering coefficients (*C*).
— In order to clarify SikuBERT’s good performance in processing ancient Chinese text from a complex network perspective, we also compare its attention networks with those generated by Chinese BERT. Interestingly, we discover that the attention networks generated by SikuBERT tend to be sparser in general, and they have a broader range for each network metric than those of Chinese BERT.
— Regarding the exploration of network evolution through downstream tasks, we find that sentence segmentation does not significantly affect network metrics, while the Part-of-speech tagging task makes attention networks sparser.

In the future, we will explore how the results of these network metrics can be applied to reflect the linguistic properties of language models for ancient Chinese exactly, and enhance the models’ performance in downstream tasks.

## Data Availability

Data and relevant code for this research work are stored in [45] and have been archived within the Zenodo repository [46].

## Footnotes

^1^

https://catalog.ldc.upenn.edu/LDC2017T14

^2^

https://huggingface.co/SIKU-BERT/sikubert

^3^

https://github.com/hsc748NLP/SikuBERT-for-digital-humanities-and-classical-Chinese-information-processing
